# Amphibians of the Reserva Ecológica Michelin: a high diversity site in the lowland Atlantic Forest of southern Bahia, Brazil

**DOI:** 10.3897/zookeys.753.21438

**Published:** 2018-04-26

**Authors:** Caio Vinícius de Mira-Mendes, Danilo Silva Ruas, Renan Manoel de Oliveira, Indira Maria Castro, Iuri Ribeiro Dias, Julio Ernesto Baumgarten, Flora Acuña Juncá, Mirco Solé

**Affiliations:** 1 Departamento de Ciências Biológicas, Universidade Estadual de Santa Cruz, Rodovia Jorge Amado, km 16, 45662-900 Ilhéus, Bahia, Brazil; 2 Programa de Pós-Graduação em Sistemas Aquáticos Tropicais, Universidade Estadual de Santa Cruz, Rodovia Jorge Amado, km 16, 45662-900 Ilhéus, Bahia, Brazil; 3 Universidade Estadual do Sudoeste da Bahia, Campus Itapetinga, Praça Primavera, 40 – Bairro Primavera, 45700-000, Itapetinga, Bahia, Brazil; 4 Programa de Pós-Graduação em Zoologia, Museu Nacional, Universidade Federal do Rio de Janeiro. Quinta da Boa Vista, São Cristóvão. 20940-040, Rio de Janeiro, RJ, Brazil; 5 Programa de Pós-Graduação em Zoologia, Universidade Estadual de Santa Cruz, Rodovia Jorge Amado, km 16, 45662-900 Ilhéus, Bahia, Brazil; 6 Departamento de Ciências Biológicas, Universidade Estadual de Feira de Santana, Avenida Transnordestina, 44036-900, Feira de Santana, Bahia, Brazil; 7 Herpetology Section, Zoologisches Forschungsmuseum Alexander Koenig, Adenauerallee 160, D-53113 Bonn, Germany

**Keywords:** Anura, biodiversity, Gymnophiona, inventory, species richness

## Abstract

An inventory of the amphibians of the Reserva Ecológica Michelin – REM in southern Bahia, Brazil is presented. Sixty-nine species were recorded during a ten-year sampling period. Amphibians were distributed in two orders (Gymnophiona and Anura), belonging to twelve families [Aromobatidae (1), Bufonidae (3), Centrolenidae (1), Craugastoridae (5), Eleutherodactylidae (3), Hemiphractidae (2), Hylidae (34), Phyllomedusidae (5) Leptodactylidae (7), Microhylidae (4), Odontophrynidae (3) and Caeciliidae (1)]. Fifty per cent of the reproductive modes known for Atlantic forest anurans were recorded. While no threatened species were found at REM, six species are classified as data deficient (DD) by the Brazilian Red List of threatened species and deserve additional attention. *Phasmahyla timbo* and *Vitreorana eurygnatha* are listed as endangered in Bahia according to the list of threatened species of the state. Despite a higher diversity of amphibians in the Atlantic forest having been reported for mountainous regions, our results revealed that amphibian richness for lowland forests is also high.

## Introduction

A rapid decline in amphibian populations has been reported worldwide over the past decades (Young et al. 2001), and currently amphibians are considered the most threatened vertebrate group on the planet (Hoffmann et al. 2010). Approximately one-third of all extant species are threatened (Stuart et al. 2008), which is a high rate in comparison to mammals (23%) and birds (12%) (Baillie et al. 2004). The major threats to the group are habitat degradation, fragmentation and destruction (Young et al. 2004, Becker et al. 2007, Loyola et al. 2007), competition from exotic species (Vredenburg 2004), infectious diseases (Daszk et al. 2003) and climate change (Carey and Alexander 2003, Pounds et al. 2006).

Within the Neotropics, Brazil harbours the largest number of described amphibian species worldwide (Segalla et al. 2016). According to the national species conservation status assessment (ICMBio 2014), only 4% of Brazilian amphibians are threatened. However, approximately 17% of Brazilian species are classified as Data Deficient (DD) (ICMBio 2014) and as there are still many gaps in distribution data, the real number of threatened species may be underestimated and much basic biogeographical work remains to be done (Brooks et al. 2004, IUCN 2008).

The Amazon and the Atlantic Forest biomes harbour the greatest species richness in Brazil (Haddad et al. 2013, Jenkins et al. 2015). Particular attention should be paid to the Atlantic Forest, which is considered one of the five most important biodiversity hotspots in the world (Myers et al. 2000, Mittermeier et al. 2011) and has one of the highest levels of amphibian richness and endemism recorded in the country (Morellato and Haddad 2000, Silva and Casteleti 2003, Haddad et al. 2013). According to Haddad et al. (2013) more than half of the country’s species occur in the Atlantic Forest, of which approximately 75% are endemic to the biome. Unfortunately, this biome has been devastated by logging, urbanization, and agricultural development (Ribeiro et al. 2011). Given the high species richness and endemism, the high degree of threat, and the lack of basic biogeographical information for most species, thorough inventories in previously unstudied areas are an essential step for planning future conservation actions (Campos et al. 2017).

Southern Bahia region is unique within the Atlantic Forest, as this area is believed to have been the largest forest refugium in the biome through the Last Glacial Maximum – LGM (Carnaval et al. 2009) and because of this is expected to harbour a rich amphibian fauna. On the other hand, recent studies have suggested that the Atlantic Forest probably expanded during the LGM onto the Brazilian continental shelf and this may have played an important role in the species diversification process (Leite et al. 2016). Until recently the only literature report on amphibian diversity from southern Bahia was a swift survey conducted by Silvano and Pimenta (2003), and although the sampling effort was limited, they recorded significant richness for several areas. During the past decade there has been an increase in studies (e.g., Dias et al. 2014a, b) which have revealed high levels of amphibian richness and endemism. They also highlight the biological importance of this region.

Despite the increasing number of publications on amphibians from Bahia over the past decades, there is still lack of data on amphibian distribution patterns. The increasing number of publications reporting the geographic distribution of several species (e.g., Camurugi et al. 2010, Dias et al. 2011, Mattedi and Pontes 2014, Dias et al. 2014a) and the description of new species corroborate this data (e.g., Napoli et al. 2011, Lourenço-de-Moraes et al. 2012, Teixeira-Jr et al. 2013, Caramaschi et al. 2013, Pontes et al. 2014, Juncá et al. 2015, Dias et al. 2017, Marciano et al. 2017, Vörös et al. 2017). Inventories increase our knowledge of amphibian community composition and allow a better understanding of species diversity patterns (Haddad 1998). These studies also allow a better assessment of species conservation status that is pivotal for developing future conservation plans (Verdade et al. 2012). Our study aims to provide an inventory of amphibian species from the Reserva Ecológica Michelin – REM, a lowland Atlantic Forest site in southern Bahia, northeastern Brazil, known as one of the most biodiverse regions of the world.

## Materials and methods

### Study area

**Figure 1.**
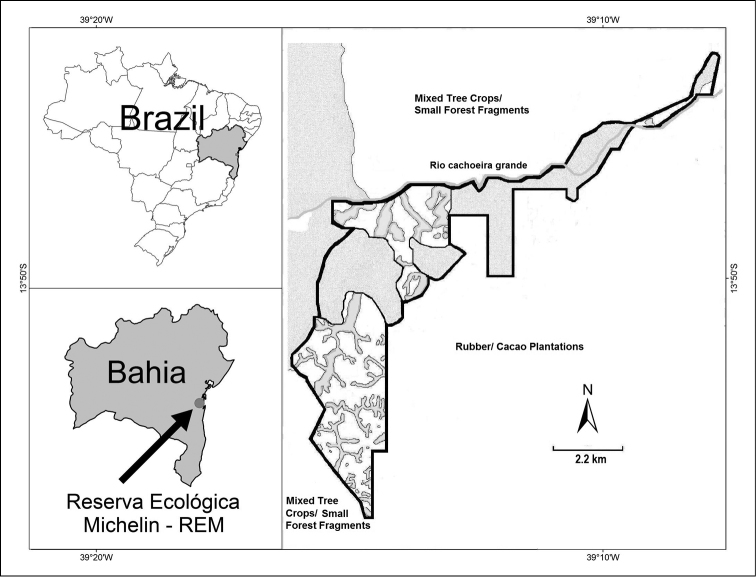
Map of the Reserva Ecológica Michelin, Bahia State, northeastern Brazil.

The study was conducted in the Reserva Ecológica Michelin – REM (Figure 1), located in the municipalities of Igrapiúna and Ituberá (13°50'S, 39°10'W), southern Bahia, northeastern Brazil. According to Veloso et al. (1991), the region is characterized as Dense Ombrophilous Lowland Forest. The 3.096 ha reserve supports 1.800 ha of lowland evergreen hill forest distributed in three main fragments (Vila 5/Pancada Grande 625 ha, Pacangê 550 ha, and Luis Inácio 140 ha). The reserve forests have a long history of human disturbance, mostly manioc farming and intensive logging, and the forest is a mosaic of secondary vegetation at different stages of regeneration and primary vegetation logged at varying intensities, with the most intact forests on the steepest slopes and ridge tops. The remainder of the reserve consists of wetlands, small forest fragments and areas with rubber plantations (*Hevea brasiliensis*) overgrown with pioneer vegetation and enriched with native forest trees (Flesher 2015). The landscape to the east supports rubber, cacao, and banana groves, while to the south, southwest and north, the landscape is one of smallholder properties of mixed tree crops and small forest fragments. A 13.000 ha forest, which is contiguous with the Pacangê forest, lies to the west. The regional landscape (1.000 km²) retains 40% forest cover and a high diversity of agroforestry systems with >60 tree crops planted (Flesher 2006). The average annual rainfall is approximately 2.000 mm with temperatures from 21.7 to 30.8° C (data from REM).

### Data sampling

Two research teams [(Universidade Estadual de Santa Cruz (UESC) and Universidade Estadual de Feira de Santana (UEFS)] have studied the amphibian community of the reserve for the past ten years. A preliminary inventory carried out between March 2007 and December 2008 revealed 48 anuran species, distributed in ten families (Camurugi et al. (2010)). Between 2010 and 2016 amphibian researchers were continuously active in the reserve, with species diversity recorded through active searching using visual and acoustic cues (Rödel and Ernst 2004) and by opportunistic encounters (i.e., along roads). Between March and December 2015, the terrestrial amphibians were studied using standard pitfall traps (Cechin and Martins 2000, Ribeiro-Júnior et al. 2011), sampling all of the reserve habitats using 24 sets of pitfall traps with 40 cm tall drift fences that included five 30-litre buckets spaced at 8-m intervals, totalling 32 m length. Pitfall traps were kept active during six nights per month, totalling a pitfalls/day effort of 7.200.

All animals were collected according to federal law (ICMBio license #13708-1) and REM protocols. Vouchers were deposited in Bahia at the Museu da Universidade Estadual de Santa Cruz (**MZUESC**) and Museu de Zoologia da Universidade Estadual de Feira de Santana (**MZFS**).

## Results

**Figure 2.**
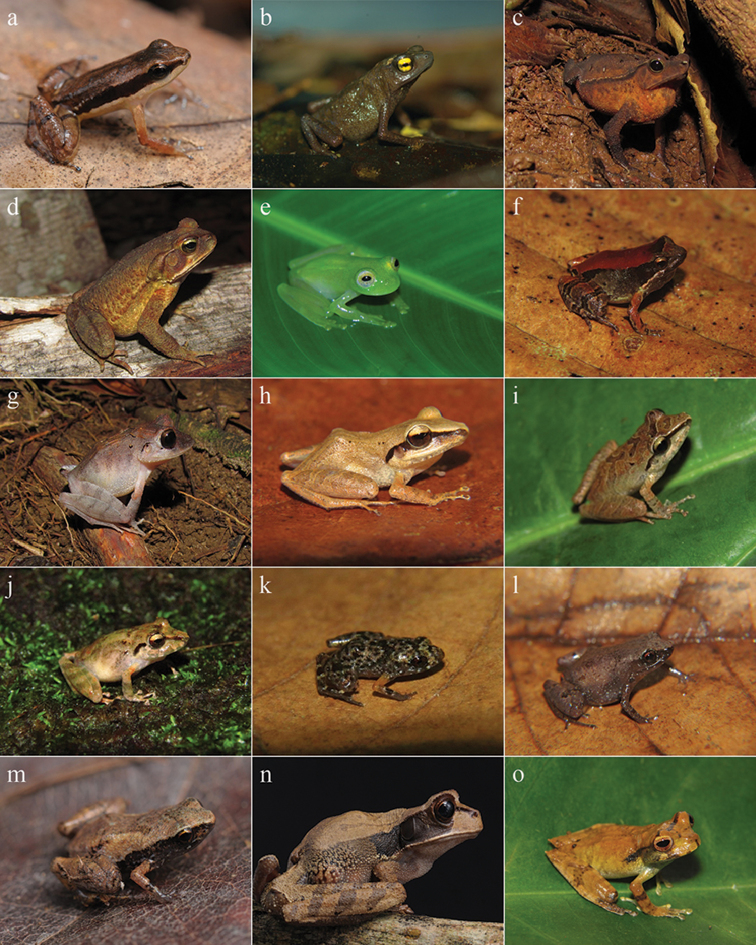
Amphibians from Reserva Ecológica Michelin, Bahia State, Northeastern Brazil. **a** *Allobates olfersioides* **b** *Frostius erythrophthalmus* **c** *Rhinella hoogmoedi* **d** *Rhinella crucifer* **e** *Vitreorana eurygnatha* **f** “*Eleutherodactylus*” *bilineatus* **g** *Haddadus binotatus* **h** *Pristimantis paulodutrai* **i** *Pristimantis* sp. **j** *Pristimantis vinhai* **k** Adelophryne cf. pachydactyla **l** *Adelophryne mucronatus* **m** *Adelophryne* sp. **n** *Gastrotheca recava* **o** *Gastrotheca* sp.

**Figure 3.**
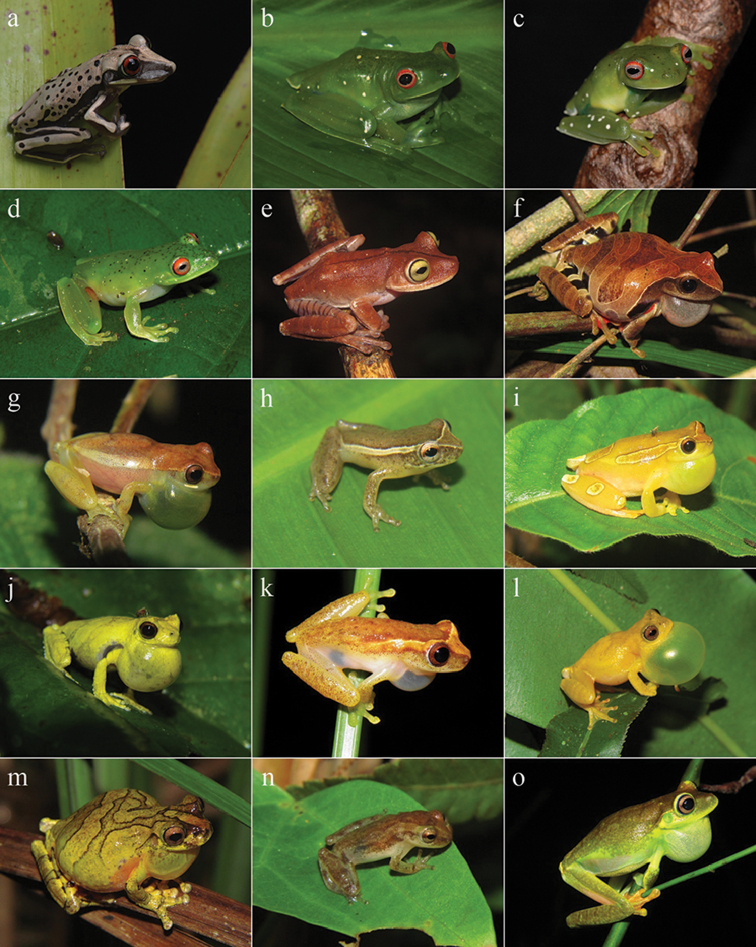
Amphibians from Reserva Ecológica Michelin, Bahia State, Northeastern Brazil. **a** *Aparasphenodon brunoi* **b** *Aplastodiscus cavicola* **c** *Aplastodiscus ibirapitanga* **d** *Aplastodiscus sibilatus* **e** *Bokermannohyla capra* **f** *Dendropsophus anceps* **g** *Dendropsophus branneri* **h** *Dendropsophus decipiens* **i** *Dendropsophus elegans* **j** *Dendropsophus giesleri* **k** *Dendropsophus haddadi* **l** *Dendropsophus minutus* **m** *Dendropsophus novaisi* **n** Dendropsophus aff. oliveirai **o** *Boana albomarginata*.

**Figure 4.**
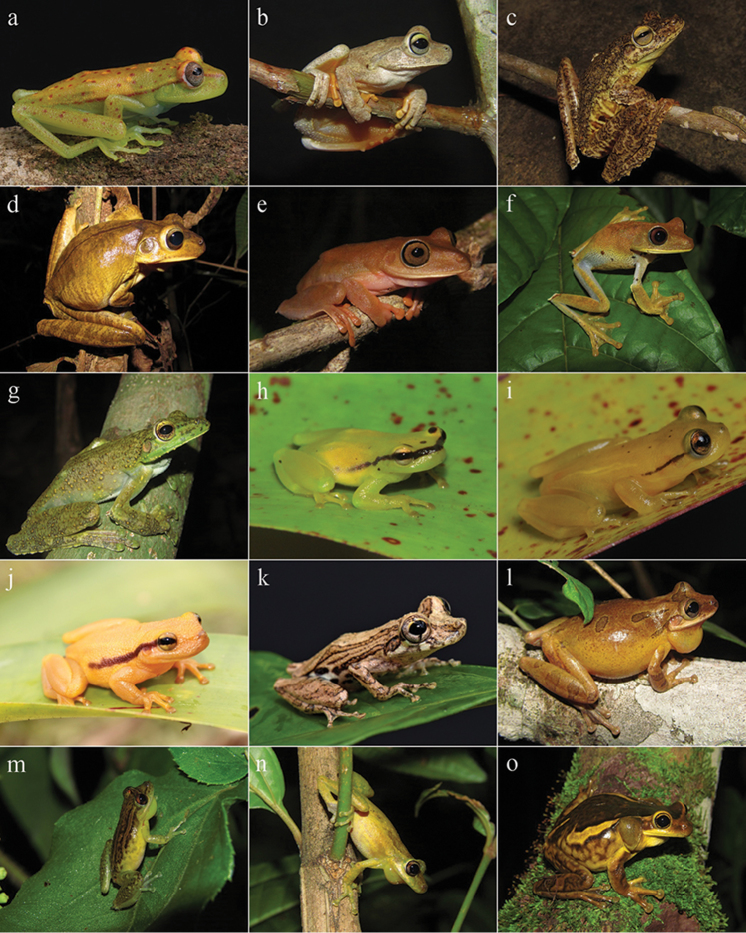
Amphibians from Reserva Ecológica Michelin, Bahia State, Northeastern Brazil. **a** *Boana atlantica* **b** *Boana crepitans* **c** *Boana exastis* **d** *Boana faber* **e** *Boana pombali* **f** *Boana semilineata* **g** *Itapotihyla langsdorffii* **h** *Phyllodytes melanomystax* **i** *Phyllodytes praeceptor* **j** *Phyllodytes* sp. **k** *Ololygon strigilata* **l** *Scinax eurydice* **m** *Scinax juncae* **n** *Scinax x-signatus* **o** *Trachycephalus mesophaeus*.

**Figure 5.**
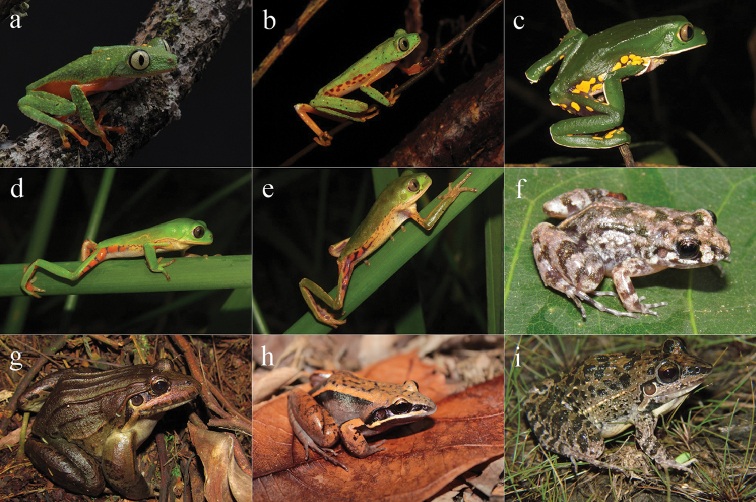
Amphibians from Reserva Ecológica Michelin, Bahia State, Northeastern Brazil. **a** *Hylomantis aspera* **b** *Phasmahyla timbo* **c** *Phyllomedusa bahiana* **d** *Pithecopus nordestinus* **e** *Pithecopus rohdei* **f** *Adenomera thomei* **g** *Leptodactylus macrosternum* **h** *Leptodactylus cupreus* **i** *Leptodactylus fuscus*.

**Figure 6.**
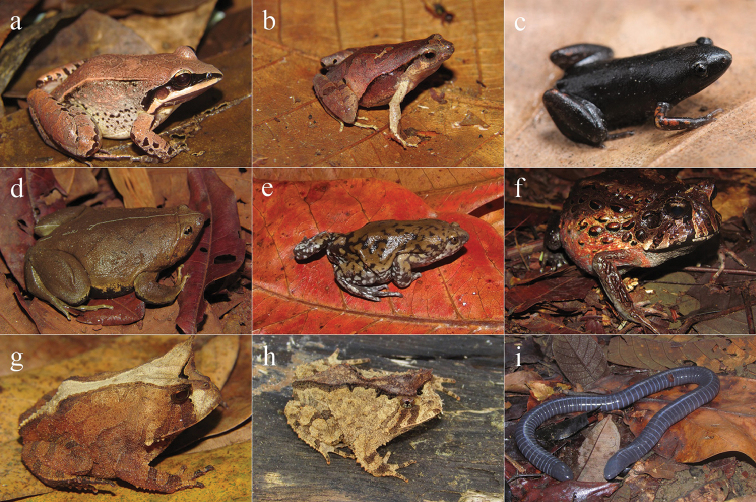
Amphibians from Reserva Ecológica Michelin, Bahia State, Northeastern Brazil. **a** *Leptodactylus mystaceus* **b** *Physalaemus camacan* **c** *Chiasmocleis cordeiroi* **d** *Stereocyclops incrassatus* **e** *Dermatonotus muelleri* **f** *Macrogenioglottus alipioi* **g** *Proceratophrys renalis* **h** *Proceratophrys schirchi* **i** *Siphonops annulatus*.

Sixty-nine species of amphibians were recorded in the REM: one species of Gymnophiona (*Siphonops annulatus* – Siphonopidae) and 68 anurans species, belonging to eleven families (Table 1; Figures 2–6). Twenty are new records for the REM, recorded since the study of Camurugi et al. (2010): *Frostius erythrophthalmus*, Adelophryne cf. pachydactyla, *Adelophryne mucronatus*, *Adelophryne* sp., *Gastrotheca* sp., *Gastrotheca recava*, *Aparasphenodon brunoi*, *Dendropsophus anceps*, *Dendropsophus decipiens*, *Boana exastis*, *Itapotihyla langsdorffii*, Phyllodytes cf. maculosus, *Phyllodytes megatympanum*, *Phyllodytes* sp. 2, *Phyllodytes wuchereri*, *Leptodactylus cupreus*, *Leptodactylus fuscus*, *Leptodactylus vastus*, *Dermatonotus muelleri* and *Siphonops annulatus*.

**Table 1.** Amphibian species found in the Reserva Ecológica Michelin, southern Bahia, Brazil. ICMBio = Instituto Chico Mendes de Conservação da Biodiversidade; **Conservation status**: VU = Vulnerable; DD = Deficient Data; LC = Least Concern. **Habitat**: F = Forest; RP = Rubber plantation. **Microhabitat**: LL = Leaf litter or understory; SV = Shrub vegetation; S = Streams; TP = Temporary ponds; PP = Permanent ponds; B = bromeliads or epiphytes; C = Canopy; F = Fossorial. **Reproductive Modes** (*sensu* Haddad et al. 2013). * = species only found in the inner forests; † = only acoustic record; # only recorded once or twice during the sampling.

Order/Family/Species	ICMBio	Habitat	Microhabitat	Reproductive modes
**ANURA**				
**Aromobatidae**				
*Allobates olfersioides* (Lutz, 1925)	VU	F	LL, S	20
**Bufonidae**				
*Frostius erythrophthalmus* Pimenta & Caramaschi, 2007*	LC	F	SV	?
*Rhinella hoogmoedi* Caramaschi & Pombal, 2006	LC	F, RP	LL, S	1
*Rhinella crucifer* (Wied-Neuwied, 1821)	LC	F, RP	LL, PP, TP	1,2
**Centrolenidae**				
*Vitreorana eurygnatha* (Lutz, 1925)*	LC	F	S	25
**Craugastoridae**				
“*Eleutherodactylus*” * bilineatus* (Bokermann, 1975)*	LC	F	LL	23
*Haddadus binotatus* (Spix, 1824)	LC	F	LL	23
*Pristimantis paulodutrai* (Bokermann, 1975)	LC	RP	SV	23
*Pristimantis* sp.*	–	F	SV	23
*Pristimantis vinhai* (Bokermann, 1975)	LC	F, RP	SV	23
**Eleutherodactylidae**				
Adelophryne cf. pachydactyla Hoogmoed, Borges & Cascon, 1994	LC	F	LL	23
*Adelophryne mucronatus* Lourenço-de-Morais, Solé & Toledo 2012*	LC	F	LL	23
*Adelophryne* sp.*	–	F	LL	23
**Hemiphractidae**				
*Gastrotheca* sp.	–	F	C	37
*Gastrotheca recava* Teixeira et al., 2012	–	F	C	37
**Hylidae**				
*Aparasphenodon brunoi* Miranda-Ribeiro, 1920*	LC	F	SV	1
*Aplastodiscus cavicola* (Cruz and Peixoto, 1985)*	LC	F	S	5
*Aplastodiscus ibirapitanga* (Cruz, Pimenta & Silvano, 2003)*	LC	F	S	5
*Aplastodiscus sibilatus* (Cruz, Pimenta & Silvano, 2003)*	LC	F	S	5
*Bokermannohyla capra* Napoli & Pimenta, 2009*	–	F	S	2
*Dendropsophus anceps* (Lutz, 1929)	LC	RB	TP	1
*Dendropsophus branneri* (Cochran, 1948)	LC	F, RP	PP, TP	1
*Dendropsophus decipiens* (Lutz, 1925)	LC	RB	PP, TP	24
*Dendropsophus elegans* (Wied-Neuwied, 1824)	LC	F, RP	PP, TP	1
*Dendropsophus giesleri* (Mertens, 1950)	LC	F, RP	TP	1
*Dendropsophus haddadi* (Bastos & Pombal, 1996)	LC	F, RP	PP, TP	24
*Dendropsophus minutus* (Peters, 1872)	LC	F, RP	TP	1
*Dendropsophus novaisi* (Bokermann, 1968)	LC	RP	TP	1
Dendropsophus aff. oliveirai (Bokermann, 1963)	LC	RB	PP, TP	1
*Boana albomarginata* (Spix, 1824)	LC	F, RP	PP, TP	1
*Boana atlantica* (Caramaschi & Velosa, 1996)	LC	F, RP	PP, TP	1,2
*Boana crepitans* (Wied-Neuwied, 1824)	LC	RB	PP	4
*Boana exastis* (Caramaschi & Rodrigues, 2003)*	LC	F	B, C, S	4
*Boana faber* (Wied-Neuwied, 1821)	LC	F, RP	PP, TP	1,4
*Boana pombali* (Caramaschi, Pimenta & Feio, 2004)	LC	F, RP	PP	1,2
*Boana semilineata* (Spix, 1824)	LC	F, RP	PP	1,2
*Itapotihyla langsdorffii* (Duméril & Bibron, 1841)	LC	F, RP	TP	1
Phyllodytes cf. maculosus Cruz, Feio & Cardoso, 2007†	DD	F	B	6
*Phyllodytes melanomystax* Caramaschi, Silva & Britto-Pereira, 1992	LC	F, RP	B	6
*Phyllodytes megatympanum* Marciano, Lantyer-Silva & Solé 2017†*	–	F, RP	B	6
*Phyllodytes praeceptor* Orrico, Dias & Marciano 2018	–	F, RP	B	6
*Phyllodytes* sp.	–	F, RP	B	6
*Phyllodytes wuchereri* (Peters, 1873)†*	LC	F	B	6
*Ololygon argyreornata* (Miranda-Ribeiro, 1926)*	LC	F	S	1
*Ololygon strigilata* (Spix, 1824)*	DD	F	S	1,2
*Scinax eurydice* (Bokermann, 1968)	LC	F, RP	PP, TP	1
*Scinax juncae* Nunes & Pombal, 2010	LC	F, RP	PP	1
*Scinax x-signatus* (Spix, 1824)	LC	F, RP	PP, TP	1
*Trachycephalus mesophaeus* (Hensel, 1867)	LC	F, RP	TP	1
**Phyllomedusidae**				
*Hylomantis aspera* (Peters, 1873)*	LC	F	TP	18
*Phasmahyla timbo* Cruz, Napoli & Fonseca, 2008*	DD	F	S	25
*Phyllomedusa bahiana* Lutz, 1925	LC	F, RP	TP	24
*Pithecopus nordestinus* (Caramaschi, 2006)	LC	F, RP	PP, TP	24
*Pithecopus rohdei* (Mertens, 1926)	LC	F, RP	PP, TP	24
**Leptodactylidae**				
*Adenomera thomei* (Almeida & Angulo, 2006)	LC	RP	LL	32
*Leptodactylus macrosternum* Miranda-Ribeiro, 1926	LC	F, RP	LL, S, PP, TP	11
*Leptodactylus cupreus* Caramaschi, Feio & São-Pedro, 2008*	DD	F	LL, TP	30
*Leptodactylus fuscus* (Schneider, 1799)	LC	RP	LL, TP	30
*Leptodactylus mystaceus* (Spix, 1824)	LC	RP	LL, TP	30
*Leptodactylus vastus* Lutz, 1930	LC	RP	LL, TP	11
*Physalaemus camacan* Pimenta, Cruz, & Silvano, 2005	LC	F, RP	LL, TP	11
**Microhylidae**				
*Chiasmocleis cordeiroi* Caramaschi & Pimenta, 2003	DD	F, RP	LL, TP	1
*Chiasmocleis crucis* Caramaschi & Pimenta, 2003	DD	F	LL, TP	1
*Stereocyclops incrassatus* Cope, 1870	LC	F, RP	LL, TP	1
*Dermatonotus muelleri* (Boettger, 1885)	LC	RP	TP	1
**Odontophrynidae**				
*Macrogenioglottus alipioi* Carvalho, 1946*	LC	F	LL, TP	1
*Proceratophrys renalis* (Miranda-Ribeiro, 1920)	LC	F	LL, S	2
*Proceratophrys schirchi* (Miranda-Ribeiro, 1937)*	LC	F	LL, S	2
**GYMNOPHYONA**				
**Caeciliidae**				
*Siphonops annulatus* (Mikan, 1820)	LC	F, RP	F	

According to the Brazilian federal list (ICMBio 2014), most of the species found in the REM are not threatened (N = 54; 78.2%), except *Allobates olfersioides* listed as Vulnerable. Approximately 9% (N = 6) are listed as data deficient (DD) and the conservation status of eight species has not yet been defined. Considering the species identified at the species level or as “cf.”, the majority (n = 44; 63.8%) are endemic to the Atlantic Forest biome, and 20.3% (n = 14) are endemic to Bahia. Two species deserve special attention: *Phasmahyla timbo* and *Vitreorana eurygnatha*, both considered endangered (EN) in the list of threatened species of the state of Bahia (Bahia 2017).

More than half of the species were recorded in lentic habitats (n = 36; 52.17%), of which 18 species were restricted to temporary ponds, four to permanent ponds, and eleven were found in both habitats (Table 1). Twenty-one species were found on leaf litter, of which two and nine, also occupied streams and temporary ponds, respectively. Eight species were found only in forest streams (*Vitreorana eurygnatha*, *Aplastodiscus cavicola*, *A. ibirapitanga*, *A. sibilatus*, *Bokermannohyla capra*, *Phasmahyla timbo*, *Ololygon argyreornata* and *O. strigilata*). Epiphytes and bromeliads were used by species of the genus *Phyllodytes*. The forest canopy was occupied primarily by *Gastrotheca* sp. and *G. recava*, frequently found between two and five meters above ground. *Boana exastis* was most frequently heard calling from bromeliads in the canopy, but was also spotted along streams. *Leptodactylus macrosternum* was the species that demonstrated the highest habitat plasticity and was found in streams, permanent and temporary ponds. Only *Siphonops annulatus* occurred in fossorial habitats. Twenty-nine species were found exclusively inside the forest, ten restricted to open areas and rubber plantations and 31 in both habitats (Table 1).

### Taxonomic and nomenclatural remarks concerning Camurugi et al. (2010)

The previous REM checklist of anurans (Camurugi et al. 2010) included several species with taxonomic uncertainties that are revised here. Most of the following species show cryptic patterns that hamper their taxonomic identification. Our analysis was based on adult and larval (whenever possible) morphological characteristics and bio-acoustic parameters.

Based on a study of the phylogenetic relationships within the anuran clade Terrarana (Canedo and Haddad 2012), the species referred as *Ischnocnema* by Camurugi et al. (2010) have been transferred to *Pristimantis*, with exception of *Ischnocnema bilineata* that was relocated as “*Eleutherodactylus*” *bilineatus* as *incertae sedis*. A recent nomenclature review carried out by Dubois (2017) showed that the genus *Hypsiboas* was erroneously recovered as a *Hyla* synonym by Faivovich et al. (2005), and that *Boana* as suggested by Gray (1825) is a valid generic name. Thus, species referred as *Hypsiboas* in Camurugi et al. (2010) have now been transferred to *Boana*. Based on molecular data, Duellman et al. (2016) resurrected the generic names *Ololygon* for the “*Scinax catharinae* clade” and *Phitecopus* for the “*Phyllomedusa hypochondrialis* group”. Thus, *Scinax strigilatus* and *S. argyreornatus* from the list by Camurugi et al. (2010) are now referred to as *Ololygon strigilata* and *O. argyreornata*. In addition, *Phyllomedusa nordestina* and *P. rohdei* are now referred to as *Pithecophus nordestinus* and *P. rohdei*. Finally, *Phyllomedusa burmeisteri* was relocated as *P. bahiana* based on molecular data sets gathered by Barth et al. (2013) and Brunes et al. (2014).

Ischnocnema aff. ramagii is re-classified as *Pristimantis* sp. This species is widely distributed in the forests of southern Bahia and is currently being described (Marciano Jr. et al. *in prep*). *Vitreorana* sp. was recorded by Camurugi et al. (2010) only in larval form, but adult males were collected and, using morphological and bio-acoustic traits, are identified as *Vitreorana eurygnatha*. Scinax aff. alter and *Chiasmocleis* sp. are re-classified as *Scinax juncae* and *Chiasmocleis crucis*, respectively. *Phyllodytes luteolus* as *Phyllodytes praeceptor* (Orrico et al. 2018). The species formerly classified as *Dendropsophus seniculus*, *Physalaemus signifer* and *Leptodactylus marmoratus*, are now classified as *Dendropsophus novaisi*, *Physalaemus camacan* and *Adenomera thomei*, respectively.

## Discussion

The present study increases the amphibian species richness of the REM in more than 30%, increasing the total number of species for the reserve from 48 to 69. The majority (n = 43, 61.4%) of the REM species are endemic to the Atlantic Forest biome (see Haddad et al. 2013). Fourteen of the 69 recorded species (*Frostius erythrophthalmus*, “*Eleutherodactylus*” *bilineatus*, Adelophryne cf. pachydactyla, *A. mucronatus*, *Gastrotheca recava*, *Hylomantis aspera*, *Bokermannohyla capra*, *Phasmahyla timbo*, *Phyllodytes praeceptor*, *Phyllodytes wuchereri*, *Ololygon strigilata*, *Physalaemus camacan*, *Chiasmocleis cordeiroi* and *C. crucis*) are also endemic to Bahia (Angulo 2008a, Juncá and Pimenta 2004, Peixoto and Pimenta 2004, Borges-Najosa and Juncá 2004, Lourenço-de-Moraes et al. 2012, Teixeira Jr. et al. 2012, Silvano and Pimenta 2010, Napoli and Pimenta 2009, Angulo 2009, Rodrigues 2006, Juncá and Silvano 2004, Angulo 2008b, Forlani et al. 2013). The results of our study expand the known distribution of *Adelophryne mucronatus* 60 km to the north (Lourenço-de-Moraes et al. 2012, Dias et al. 2014a). Two species were recorded by collecting only a single individual per species: *Aparasphenodon brunoi*, a bromeliad species, which had its distribution recently increased from the municipality of Una to the REM (Ruas et al. 2013), and *Dermatonotus muelleri*, which is a species typically found in open areas..

Although no species has been considered threatened by ICMBio (2014), special attention should be paid to *Allobates olfersioides*. In a taxonomic review of *Allobates* from the Atlantic Forest, Verdade and Rodrigues (2007) synonymized the four previously recognized species *Allobates olfersioides* (Lutz, 1925), *A. capixaba* (Bokermann, 1967), *A. carioca* (Bokermann, 1967) and *A. alagoanus* (Bokermann, 1967) with *A. olfersioides*. However, in a recent assessment of threatened Brazilian amphibians (Haddad et al. 2016), specialists suggested that only populations from Rio de Janeiro should be recognized as *A. olfersioides* (U. Caramaschi pers. comm.) while the populations from Bahia should be assigned to *A. capixaba* or *A. alagoanus* and classified as Data deficient (DD). A recent acoustic analysis of the Atlantic Forest *Allobates* agrees with the suggestion of Haddad et al. (2016), arguing that according to the advertisement calls, populations from Bahia can be attributed to *A. capixaba* or even represent a new species (Forti et al. 2017). However, due to the lack of a recent taxonomic analysis, the species from REM is assigned to *A. olfersioides* following Verdade and Rodrigues (2007).

The two species classified as endangered (EN) in the list of threatened species of the state of Bahia (Bahia, 2017) occur in streams in the interior of forest fragments. *Phasmahyla timbo* is restricted to the state of Bahia and known only from the type locality in Serra do Timbó, municipalities of Amargosa and Santa Terezinha (Cruz et al. 2008) and from the Reserva Ecológica Michelin. *Vitreorana eurygnatha* has been reported from Amargosa (Freitas et al. 2007), Mata de São João, Jandaíra (Tinoco et al. 2008), Camacan and Almadina (Dias et al. 2014a, b), and now also from Igrapiúna, at REM.

Seven species (10.1%) are listed without a specific name or were classified as similar with other species. The formal descriptions of some of them, like *Adelophryne* sp. have already been submitted for publication. Our results add basic data on the distribution of amphibians from Bahia, and corroborate the data presented by other recent inventory studies conducted in the state (Dias et al. 2014a, b). The amphibian richness from the REM is the second-highest reported from Bahia, ranking only behind that of the RPPN Serra Bonita (80 ssp.), located along an altitudinal gradient of 200 to 950 m a.s.l. (Dias et al. 2014a). Most other Atlantic Forest sites with high amphibian diversity, such as Santa Tereza municipality (92 ssp.), the Parque Natural Municipal Nascentes de Paranapiacaba (80 ssp.), Estação Ecológica de Boracéia (67 ssp.) and Parque Estadual Carlos Botelho (65 ssp.), represent mountainous areas (Heyer et al. 1990, Forlani et al. 2010, Almeida et al. 2011, Trevine et al. 2014). Over the altitudinal gradients, changes in biotic and abiotic features increase the availability of microenvironments which are believed to promote greater species diversity. Vasconcelos et al. (2010) conducted a review in various mountainous ranges in Brazil and found that the increasing amphibian richness is related to the degree of the altitude gradient. Unlike these areas, the REM is inserted in a region classified as Dense Ombrophilous Lowland Forest (Veloso 1991), with maximum altitudes reaching 393 m above sea level.

Despite the lack of distinct altitudinal gradients, the REM is located close to 13.000 ha of forest and nested in a climatically stable region, with high moisture and availability of breeding sites. These diverse habitats provide a high diversity of breeding sites such as temporary and permanent ponds, streams, bromeliads, epiphytes and a dense leaf litter layer. This habitat heterogeneity, high temperatures and rainfall throughout the year create a hot and moist environment, which likely explains the expressive number of species recorded. Additionally, the high abundance of breeding habitats satisfies the reproductive requirements of a large number of species. According to Duellman (1988) reproductive modes play an important role in understanding anuran species diversity. Currently, there are 27 reproductive modes recognized for Atlantic Forest amphibians (Haddad et al. 2013). According to Haddad and Prado (2005), the high diversity of reproductive modes observed for the Atlantic Forest is the result of a successful utilization of the diverse humid microhabitats present in the biome. Fourteen out of the 27 reproductive modes reported for the Atlantic Forest amphibians (~52%) were recorded at the REM.

Another important factor that likely affected the results was the sampling effort. With approximately ten years of sampling this study has the highest sampling effort for northeastern Brazil to date. Long sampling periods are essential for understanding community structure and are necessary for reaching accurate values of diversity for an area. Although the present study shows an increase of 21 amphibian species in comparison to the previous study (Camurugi et al. 2010), we believe that further fieldwork may still reveal new species for the REM. Highest amphibian diversity areas in the Atlantic forest were also the result of long sampling periods. Despite the long sampling periods new species are still found in well sampled regions, as the case of *Adelophryne glandulata* and *Dendropsophus bromeliaceus*, recently described from Santa Tereza municipality (Lourenço-de-Moraes et al. 2014, Ferreira et al. 2015). Most long-term amphibian studies have been undertaken in southeastern Brazil, resulting in a much better comprehension of the amphibians of this region within the domain (Rossa-Feres et al. 2011, Campos et al. 2014). On the other hand, the recent increase in studies from other regions of the country (e.g., southern Bahia) is expanding our knowledge for these regions (Dias et al 2014a, b, present study).

Several attempts to understand the processes responsible for the high levels of species diversification of the Atlantic Rainforest have been undertaken. The consensus is that none seem to have acted isolated. The high diversity and endemism of species in southern Bahia has been associated with climate stability and forest conditions during glacial periods (Carnaval et al. 2009). According to these authors, this region was a large Pleistocene climatic refugium for amphibians. Another view of a recent research shows that suitable climatic conditions onto the emerged continental shelf probably expanded the Atlantic Forest during the last glacial period (Leite et al. 2016). Thereby, species could have expanded their distributions during the last glacial period. Thus, these long-term biogeographical processes would have promoted a high level of speciation in southern Bahia. Given the high levels of richness and endemism of amphibians in southern Bahia, future inventories in still un-sampled regions of southern Bahia are expected to recover a high diversity of amphibians, and may result in the discovery of additional new species and expand the ranges of already known species.
